# Geochronological and geochemical data from fracture-filling calcites from the Lower Pedraforca thrust sheet (SE Pyrenees)

**DOI:** 10.1016/j.dib.2020.105896

**Published:** 2020-06-20

**Authors:** David Cruset, Irene Cantarero, Antonio Benedicto, Cédric M. John, Jaume Vergés, Richard Albert, Axel Gerdes, Anna Travé

**Affiliations:** a Departament de Mineralogia, Petrologia i Geologia Aplicada, Facultat de Ciències de la Terra, Universitat de Barcelona, (UB), Martí i Franquès s/n, 08028 Barcelona, Spain; b UMR Geops, Université Paris Sud, 91405 Orsay, France; c Department of Earth Science and Engineering, Imperial College London, SW7 2BP, United Kingdom; d Group of Dynamics of the Lithosphere (GDL). Institut de Ciències de la Terra Jaume Almera, ICTJA-CSIC, Lluís Solé i Sabaris s/n, 08028 Barcelona, Spain; e Department of Geosciences, Goethe-University Frankfurt, 60438 Frankfurt am Main, Germany; f Frankfurt Isotope and Element Research Center (FIERCE), Goethe-University Frankfurt, Frankfurt am Main, Germany

**Keywords:** Fluid flow, SE Pyrenees, U-Pb dating, Fluid geochemistry

## Abstract

U-Pb dating using laser ablation-inductively coupled plasma mass spectrometry (LA-ICP-MS), δ^13^C, δ^18^O, clumped isotopes and ^87^Sr/^86^Sr analysis, and electron microprobe have been applied to fracture-filling calcites and host carbonates from the Lower Pedraforca thrust sheet, in the SE Pyrenees. These data are used to determine the type and origin of migrating fluids, the evolution of the palaeohydrological system and timing of fracturing during the emplacement of this thrust sheet, as described in the article “From hydroplastic to brittle deformation: controls on fluid flow in fold and thrust belts. Insights from the Lower Pedraforca thrust sheet (SE Pyrenees)” – Marine and Petroleum Geology (2020). The integration of these data is also used to compare the fluid flow evolution of the Southern Pyrenees with that of other orogens worldwide and to generate a fluid flow model in fold and thrust belts. At a more local scale, the U-Pb dataset provides new absolute ages recording the deformation in the Lower Pedraforca thrust sheet, which was previously dated by means of indirect methods such as biostratigraphy of marine sediments and magnetostratigraphy of continental deposits.

## 1. Value of the data

• This data set provides new insights of the relationship between fluid flow and deformation in the SE Pyrenees during a particular period of their tectonic history.
• This data is of interest for geoscientists studying the relationships between fluid flow and deformation in fold and thrust belts and those working in the geochronology of the SE Pyrenean deformation.
• This data and their comparison with datasets from other orogens worldwide are useful to perform general models of fluid flow in fold and thrust belts.
• The clumped isotopes data provide evidence of the dominant thermal regime in the Lower Pedraforca thrust sheet during deformation, which can be used as analogue for similar deformed areas that are under geothermal exploration.

## 2. Data description

Data were obtained from 57 representative samples of six different host rocks and eight types of calcite cement (Cc1 to Cc8) precipitated in seven types of fractures (F1 to F7). The location of these samples is shown in Table 1. The main features of fractures and calcite cement and host rocks are described in [1].

**Table 1:** Location of the studied samples in [1].

Sample	Locality	Description	Latitute	Longitude
Est1	EST	F7	42° 15′ 27.69″N	1° 41′ 14.68″E
Est2	EST	F7	42° 15′ 27.65″N	1° 41′ 15.51″E
Est3	EST	F7	42° 15′ 28.66″N	1° 41′ 13.73″E
G1	G1	F6	42° 7′ 54.99″N	1° 51′ 43.60″E
G2	G1	F5	42° 7′ 55.91″N	1° 51′ 44.91″E
G3	G1	F6	42° 7′ 55.99″N	1° 51′ 45.21″E
G4	G1	F5	42° 7′ 56.39″N	1° 51′ 47.43″E
G5	G1	F5	42° 7′ 57.85″N	1° 51′ 48.84″E
G6	G1	F5	42° 8′ 7.25″N	1° 51′ 55.40″E
G7	G1	Vug Porosity	42° 8′ 7.27″N	1° 51′ 55.29″E
G8	G1	F4	42° 7′ 55.84″N	1° 51′ 48.30″E
G9	G1	F4	42° 7′ 55.71″N	1° 51′ 48.23″E
G10	G1	F4	42°10′ 2.98″N	1° 53′ 15.93″E
G11	G1	F4	42° 8′ 1.50″N	1° 51′ 54.78″E
G12	G2	F3	42° 14′ 17.55″N	1° 51′ 56.96″E
G13	G2	F4	42 °14′ 17.35″N	1° 51′ 56.90″E
G14	G2	F5	42° 14′ 17.20″N	1° 51′ 56.53″E
G15	G2	F3	42° 14′ 16.60″N	1° 51′ 55.84″E
G16	G2	F3	42° 14′ 16.52″N	1° 51′ 55.36″E
PEG1	PEG	F4	42° 9′ 52.93″N	1° 45′ 37.58″E
PEG2	PEG	F4	42° 9′ 52.91″N	1° 45′ 37.80″E
PEG3	PEG	F4	42° 9′ 52.91″N	1° 45′ 37.80″E
PEG4	PEG	F4	42° 9′ 52.91″N	1° 45′ 37.80″E
PEG5	PEG	Palaeocene host rock	42° 9′ 53.25″N	1° 45′ 37.33″E
Q1	Q	F3	42° 6′ 37.71″N	1° 49′ 28.34″E
Q2	Q	F3	42° 6′ 37.52″N	1° 49′ 28.16″E
Q3	Q	F3	42° 6′ 37.47″N	1° 49′ 27.98″E
Q4	Q	F2	42° 6′ 37.51″N	1° 49′ 27.55″E
Q5	Q	F4	42° 6′ 36.18″N	1° 49′ 27.01″E
Q6	Q	F2	42° 6′ 35.92″N	1° 49′ 26.89″E
Q7	Q	F5	42° 6′ 38.18″N	1° 49′ 26.99″E
Q8	Q	Vug Porosity	42° 6′ 37.44″N	1° 49′ 28.18″E
Q9	Q	F2	42° 6′ 35.92″N	1° 49′ 27.26″E
Q10	Q	F3	42° 6′ 28.49″N	1° 49′ 34.02″E
Q11	Q	F3	42° 6′ 26.07″N	1° 49′ 35.81″E
Q12	Q	F3	42° 6′ 22.29″N	1° 49′ 25.84″E
Q13	Q	F2	42° 6′ 54.44″N	1° 50′ 14.56″E
Q14	Q	F3	42° 6′ 54.63″N	1° 50′ 14.52″E
Q15	Q	F5	42° 6′ 53.62″N	1° 50′ 16.15″E
Q16	Q	F3	42° 6′ 54.58″N	1° 50′ 14.30″E
Q17	Q	F3	42° 6′ 56.97″N	1° 50′ 28.89″E
Q18	Q	F3	42° 6′ 58.12″N	1° 50′ 30.16″E
Q19	Q	F5	42° 7′ 0.54″N	1° 50′ 32.33″E
Q20	Q	Vug Porosity	42° 7′ 0.91″N	1° 50′ 32.96″E
Q21	Q	F4	42° 7′ 0.63″N	1° 50′ 33.11″E
Q22	Q	F5	42° 7′ 3.68″N	1° 50′ 38.20″E
Q23	Q	F5	42° 7′ 1.49″N	1° 50′ 36.12″E
Q24	Q	F4	42° 7 '0.79″N	1° 50′ 33.94″E
Q25	Q	F4	42° 7′ 3.33″N	1° 50′ 14.23″E
Q26	Q	F4	42° 7′ 2.94″N	1° 50′ 14.80″E
Q27	Q	F3	42° 6′38.01″N	1° 49′ 26.53″E
Q28	Q	F3	42° 7′ 0.15″N	1° 50′ 31.65″E
Q29	Q	F4	42° 7′ 5.38″N	1° 50′ 38.37″E
Q30	Q	F4	42° 7′ 5.40″N	1° 50′ 38.26″E
Q31	Q	F4	42° 7′ 5.40″N	1° 50′ 38.26″E
Q32	Q	F1	42° 6′ 28.07″N	1° 49′ 34.68″E
Q33	Q	F3	42° 6′ 26.25″N	1° 49′ 35.67″E

The U-Pb dating of calcite cement Cc3, Cc4, Cc6, Cc7, and Cc8 was applied by LA-ICPMS on 12 samples. From these, nine dates ranging from 47.9 ± 1.3 to 42.3 ± 0.8 Ma were obtained from 240 spot analyses (Table 2). Tera-Wasserburg plots in Fig. S1 from the supplementary material of [1] show the presence of variable amounts of common and radiogenic lead that correlate well with the Pb-U, yielding for most samples well-defined regression lines with MSWD (mean square weighted deviation) of < 2. Uncertainties (2σ) of most analysis spots are low (small circles), and the uncertainties (2σ) of the lower intercept ages range from 0.7 to 2.0 Ma (1.5 and 6.6%). Samples for which the U-Pb dating method failed are characterized by high common lead and low uranium contents. The raw data of U-Pb analyses are presented in the supplementary material of [1].

**Table 2:** U and Pb contents of calcite cement Cc3, Cc4, Cc6, Cc7 and Cc8.

Calcite cement	Number of spots	Samples		U (ppm)	Pb (ppm)
Cc3	51	Q3–1	Min.	0.005	<d.l.
		Q27	Max.	0.813	0.019
			Mean	0.24	0.004
Cc4	53	Q11	Min.	0.022	0.001
		Q33	Max.	1515	0.217
			Mean	50	0.016
Cc6	59	Q24	Min.	0.077	0.001
		Q29	Max.	3253	1388
			Mean	577	23.67
Cc7	56	G3	Min.	0.084	0.002
		G3b	Max.	6502	0.35
			Mean	1099	0.045
Cc8	21	EST2	Min.	0.023	0.01
			Max.	1817	0.144
			Mean	264	0.04

From the LA-ICPMS method used for U-Pb dating, the U and Pb content of calcite cement Cc3, Cc4, Cc6, Cc7, and Cc8 also were measured (Table 2). For Cc3, the U and Pb content range from 0.005 to 0.813 ppm and from below the detection limit to 0.019 ppm, respectively (*n* = 51). The U content for Cc4 range from 0.022 to 1515 ppm and the Pb content from 0.001 to 0.217 ppm (*n* = 53). For Cc6, the U and Pb content range from 0.077 to 3253 ppm and from 0.001 to 1388 ppm, respectively (*n* = 59). The U content for Cc7 range from 0.084 to 6502 ppm and the Pb content from 0.002 to 0.35 ppm (*n* = 56). For Cc8, the U and Pb content range from 0.023 to 1817 ppm and from 0.01 to 0.144 ppm, respectively (*n* = 21).

The δ^13^C and δ^18^O composition of calcite cement Cc1 to Cc8 is already presented in [1]. Mean value and standard deviation of these results is presented in Table 3. Cc2 has the lowest mean δ^13^C (−8.5‰ VPDB), whereas Cc5 has the highest value (+1.3‰ VPDB). Regarding to the δ^18^O, Cc4 and Cc7 have the lowest and highest mean values, respectively (−4.6 and −11.1‰ VPDB). For Cc2 the δ^13^C and δ^18^O present standard deviations of 1.6 and 0.4‰ VPDB, respectively (*n* = 2). For Cc3 the δ^13^C and δ^18^O present a standard deviation of 5 and 1‰ VPDB, respectively (*n* = 9). For Cc4 the standard deviation is 0.8‰ VPDB for the δ^13^C and 1.7‰ VPDB for the δ^18^O (*n* = 6). For Cc5, the δ^13^C and δ^18^O present standard deviations of 0.2 and 0.5‰ VPDB, respectively (*n* = 3). The standard deviation for Cc6 is 4‰ VPDB for δ^13^C and 1.4‰ VPDB for δ^18^O (*n* = 31). In Cc7, the calculated standard deviation is 0.4‰ VPDB for δ^13^C and 0.8‰ VPDB for δ^18^O (*n* = 3). For Cc8, the δ^13^C has a standard deviation of 0.02‰ VPDB, whereas for the δ^18^O this value is of 0.6‰ VPDB (*n* = 2).

**Table 3:** Mean value and standard deviation of the δ^18^O and δ^13^C in ‰ VPDB of the calcite cements Cc2, Cc3, Cc4, Cc5, Cc6, Cc7 and Cc8 and precipitated in the Lower Pedraforca thrust sheet. Calculations were not made for Cc1 because only one analysis could be done.

Cement	n	δ^13^C ‰ VPDB	Standard deviation	δ^18^O ‰ VPDB	Standard deviation
Cc2	2	−8.5	1.6	−5.6	0.4
Cc3	9	−5.9	5	−5.3	1
Cc4	6	−5.2	0.8	−4.6	1.7
Cc5	3	+1.3	0.2	−5.1	0.5
Cc6	31	−1.6	4	−8.1	1.4
Cc7	3	−1.3	0.4	−11.1	0.8
Cc8	2	−0.25	0.02	−7.1	0.6

Clumped isotopes and ^87^Sr/^86^Sr results are presented and interpreted in [1]. Clumped isotopes thermometry was only applied to calcite cement Cc3 and Cc6. The measured temperatures are 69.1 ± 5.3 °C for Cc3 and 74.2 ± 4 °C for Cc6. The calculated δ^18^O_fluid_ for these cements are + 5.4 ± 0.9‰ VSMOW for Cc3 and + 5.1 ± 0.7‰ VSMOW for Cc6. Calculations were made using the measured clumped isotopes temperatures, the δ^18^O of calcite cements and the formula of [2]. The ^87^Sr/^86^Sr was measured in calcite cement Cc3, Cc5 and Cc6 and of one sample of Upper Cretaceous limestones. Analyses provide ratios from 0.707817 to 0.708230.

The Mg, Sr, Fe and Mn content in ppm of calcite cement Cc1, Cc3, Cc4, cc5, and Cc6 was determined through 170 electron microprobe spot analyses presented in [1]. The mean content and the standard deviation of the elemental composition of each cement is presented in Table 4. Cc5 has the lowest Mg mean content (1790 ppm), whereas Cc1 has the lowest value (3999 ppm). For Sr, Cc3 and Cc5 have the lowest and highest mean, respectively (705 and 1040 ppm). Cc5 has the highest Fe mean content (1868 ppm), whereas for Cc4 all the values are below de detection limit. Regarding to the Mn content, all the studied calcite cements have similar values ranging between 207 and 233 ppm. For Mg, Cc1 shows with 1883 ppm the highest deviation and Cc5 the lowest deviation with 488 ppm. For Sr, Cc1 also has with 678 ppm the highest variation, whereas Cc3 to Cc2 show similar values (between 183 and 392 ppm). Regarding to the Fe content, Cc5 shows the highest deviation with values of 758 ppm, whereas Cc3 only shows a deviation of 125 ppm. Standard deviation in Cc4 was not calculated because all their values are below detection limit. Mn contents show deviations of around 60 ppm for Cc1, Cc5 and Cc5, whereas for Cc3 and Cc4 the deviation is 88 and 36 ppm, respectively.

**Table 4:** Mean content in ppm and calculated standard deviations for Mg, Sr, Fe and Mn of calcite cements Cc1, Cc3, Cc5 and Cc6 precipitated in locality Q. n represent the number of spots of analysis.

Cement	N	Mg (ppm)	Std. Dev.	Sr (ppm)	Std. Dev.	Fe (ppm)	Std. Dev.	Mn (ppm)	Std. Dev.
Cc1	38	3999	1883,13,965	974	678,305,284	567	430,486,686	227	60,5,155,831
Cc3	29	2369	894,033,411	522	183,440,017	225	124,834,634	233	87,9,163,463
Cc4	20	2394	1263,36,851	705	323,446,028	–	–	207	36,0,701,158
Cc5	37	1790	487,890,479	1040	392,217,244	1868	758,133,751	216	64,3,943,173
Cc6	47	2389	959,542,576	784	245,672,521	1436	510,844,098	209	62,1,912,234

## 3. Experimental design, materials and methods

The methods and analytical protocols followed for the geochronological and geochemical analysis of samples is the same described in [1]. Prior to these analyses, petrographic observations of 57 polished thin sections made from host rocks and fracture-filling calcite cement were made using optical and cathodoluminescence microscopy. A CL Technosyn cathodoluminescence device Model 8200 MkII at 15–18 kV operating conditions and 350 μA gun current was used to distinguish the different types of cement.

U-Pb dates were acquired using laser ablation-inductively coupled plasma mass spectrometry (LA-ICPMS) at FIERCE (Frankfurt Isotope and Element Research Center, Goethe Universität). Each analysis consisted of 18 s of background acquisition followed by 18 s of sample ablation and 25 s of washout. Prior to analysis, each spot was pre-ablated with 8 laser pulses to remove surface contamination. Soda-lime glass NIST SRM 614 was used as a primary reference together with three carbonate reference materials. Raw data were corrected offline using an in-house VBA spreadsheet program [3, 4]. Due to the carbonate matrix, additional offset factors were applied, which were determined using WC-1 carbonate reference material [5]. A stromatolitic limestone from the Cambrian-Precambrian boundary in South-Namibia (here called NAMA) was analysed and used as a secondary in-house RM for quality controlData were plotted in Tera-Wasserburg diagrams and ages were calculated as lower intercepts using Isoplot 3.71 [6]. All uncertainties are reported at the 2σ level.

For δ^13^C and δ^18^O analysis of calcite cement and carbonate host rocks, a 400 μm-thick dental drill was employed to extract 60 ± 10 μg of carbonate powder from trims. The calcite powder was reacted with 100% phosphoric acid for 2 min at 70 °C. The resultant CO_2_ was analysed using an automated Kiel Carbonate Device attached to a Thermal Ionization Mass Spectrometer Thermo Electron (Finnigan) MAT-252. The International Standard NBS-18 and the internal standard RC-1, traceable to the International Standard NBS-19, were used for calibration. The results were corrected with respect to the VPDB (Vienna Pee Dee Belemnite) standard. Standard deviation is ± 0.02‰ for δ^13^C and ± 0.05‰ for δ^18^O.

For clumped isotopes thermometry, 2–3 mg aliquots of powdered carbonates were measured with the Imperial Batch Extraction system (IBEX), an automated line developed at Imperial College of London. Each sample was dropped in 105% phosphoric acid at 90 °C and reacted for 30 min. The reactant CO_2_ was separated using a poropak-Q column and transferred into the elbows of a Thermo Scientific MAT 253 mass spectrometer (Thermo Fisher GmbH, Bremen, Germany). The post-acquisition processing was completed with a software for clumped isotope analysis named Easotope [7]. ∆_47_ values were corrected for isotope fractionation during phosphoric acid digestion employing the method of [8]. The data were also corrected for non-linearity applying the heated gas method [9] and projected into the absolute reference frame of [10]. Samples were measured three times and the average result was converted to temperatures using the calibration method of [11]. Calculated δ^18^O values of the fluid are expressed in ‰ with respect to the VSMOW standard (Vienna Standard Mean Ocean Water).

For ^87^Sr/^86^Sr analysis, 100% carbonate samples are dissolved in 5 ml of 10% acetic acid and introduced in an ultrasonic bath for 15 min. After this time, samples are dried after being centrifuged during 10 min at 4000 rpm. The remaining sample is digested in 1 ml of 3 M HNO_3_ and dried. Finally, the resultant product is digested again in 3 ml of 3 M HNO_3_ and introduced in chromatographic columns. The chromatographic separation of Sr was done using an extraction resin type SrResinTM (Trisken International) (crown-ether (4.4′ (5′)-di-t-butylcyclohexano-18-crown-6). The Sr is recovered with HNO_3_ 0.05 M as eluent. The fraction where Sr is concentrated is dried, charged on a Re single filament with 1 μl of H_3_PO_4_ 1 M and 2 μl of Ta_2_O_5_ and analysed on a TIMS-Phoenix mass spectrometer. The method of acquisition of data consists of dynamic multicollection during 10 blocks of 16 cycles each one, with a beam intensity for the ^88^Sr mass of 3 V. Analyses were corrected for possible interferences of ^87^Rb. The ^87^Sr/^86^Sr ratios are normalized with respect to the measured mean value of the ratio ^86^Sr/^88^Sr = 0.1194 in order to correct possible mass fractionation during filament charge and instrumental analyses. During sample analysis, the isotopic standard NBS-987 was measured seven times obtaining a mean value of 0.710247 and a standard deviation 2σ of 0.000008. The precision of the analytical standard error or internal precision is 0.000009. The analytical errors referred to 2σ confidence levels in the ^87^Sr/^86^Sr ratio are 0.000003.

Carbon-coated polished thin sections were used to analyse major, minor, and trace element concentrations on a CAMECA SX-50 electron microprobe. The microprobe was operated using 20 kV of excitation potential, 15 nA of current intensity and a beam diameter of 10 µm. Analytical standards included natural silicates, carbonates and oxides as follows: calcite (Ca), dolomite (Mg), Fe2O3 (Fe), rhodonite (Mn) and Celestite (Sr). The detection limits were 135 ppm for Mn, 127 ppm for Fe, 101 ppm for Ca, 146 ppm for Na, 180 ppm for Mg, and 390 ppm for Sr. Precision on major element analyses averaged 0.64% standard error at 2σ confidence levels.

## Declaration of Competing Interest

The authors declare that they have no known competing financial interests or personal relationships which have, or could be perceived to have, influenced the work reported in this article.
